# Quantifying assessment of American ginseng (*Panax quinquefolius L.*) main root bruising based on FEM

**DOI:** 10.3389/fpls.2025.1575019

**Published:** 2025-06-06

**Authors:** Pengcheng Jia, Guixuan Zhu, Junzhi Chen, Dong Wang, Han Tang, Jinwu Wang

**Affiliations:** College of Engineering, Northeast Agricultural University, Harbin, China

**Keywords:** pendulum test, collision, LS-DYNA, bruise volume, bruise resistance index

## Abstract

Precise numerical simulation technology enabled the capture of subtle deformations in the American ginseng internal structure, allowing for an accurate assessment of bruise extent. In this study, a bilayer constitutive model of the American ginseng main root was developed through reverse engineering. The model accuracy was validated by velocity, exterior bruise area, and internal bruise area, with the highest velocity error being 3.8 %. Experiments analyzed the dynamic mechanical response of the American ginseng main root during collisions at various drop angles (30°, 50°, 70°, and 90°) and with different contact materials (steel, rubber, wood, and PVC). The effects of various collision conditions on bruise volume and bruise resistance index of the American ginseng main root was examined. The results demonstrated that a greater drop angle results in a larger bruise volume. The maximum bruise volumes were 3583.26 mm^3^ for steel and 3062.19 mm^3^ for wood. At a 30° drop angle, the bruise resistance index of the American ginseng main root was 59.14 mJ mm^-3^ for rubber and 40.89 mJ mm^-3^ for wood. At a 90° drop angle, the bruise resistance index was 45.72 mJ mm^-3^ for rubber and 35.38 mJ mm^-3^ for wood. At the same drop angles, the bruise resistance index of the American ginseng main root was consistently higher when rubber was used as the contact material. This study provides a scientific basis for the design and optimization of harvesting machinery and its key components, with the objective of effectively controlling the American ginseng bruising problem.

## 1 Introduction

American ginseng possesses significant medicinal value and is widely utilized in traditional Chinese medicine. Due to its high moisture content at harvest, American ginseng is susceptible to collisions with mechanical components during transportation and storage. Bruising from collisions can cause American ginseng to deteriorate and decay, negatively impacting its economic value and product quality. Therefore, conducting an in-depth study on American ginseng bruising during harvesting is crucial for optimizing mechanized harvesting methods.

When external forces applied to agricultural products exceed their biological yield limit, it caused cell structure rupture and bruising (Miraei Ashtiani et al., 2019). This not only diminishes the appearance and quality of agricultural products but also increased the rate of spoilage and decomposition, raising concerns over product security (Yousefi et al., 2016). Some researchers investigated various techniques to study bruising in agricultural products during harvesting. Common methods included visual inspection, image processing, and infrared spectroscopy to assess the bruise susceptibility and bruise resistance indices of agricultural products (He et al., 2015). Quantifying agricultural product bruising was typically achieved by measuring or estimating bruise volume (Li and Thomas, 2014). Methods for measuring bruised areas included directly using calipers (Jiménez-Jiménez et al., 2013), capturing and analyzing images with a machine vision system (Komarnicki et al., 2017; Zhou et al., 2016), or calculating bruise volume using the “closed volume method” (Holt and Schoorl, 1982). These methods effectively quantified bruise in agricultural products. However, these methods struggled to accurately assess the degree of bruise in the internal tissues of agricultural products, and their detection and judgment relied heavily on manual experience. Some studies tracked the distribution of contact stress to provide detailed mechanical data, capturing complex behaviors and stress concentration areas, which more accurately predicted agricultural product bruising. For example, Lewis et al. (2008) used haptic detection software to test pressure dispersion during fruit loading, obtaining data on bruised areas. Herold et al. (2001) used Tekscan 5051 flexible grid pressure sensors and ultrasound technology to measure and analyze quasi-static compression contact stress distribution in fruits, thereby predicting bruising. Wu et al. (2012) used pre-scaled sensing films to measure contact stress distribution in fruits during drop collisions, achieving reliable predictions of mechanical bruising. However, equipment complexity, high technical barriers, and costs limit the feasibility of large-scale application in agricultural product bruise prediction. Additionally, dealing with complex fruit shapes and diverse bruise patterns led to issues with measurement accuracy and consistency (Keresztes et al., 2017). These methods posed challenges to achieving efficient and accurate bruise assessment during harvesting. In contrast, numerical simulation technology offered significant advantages in this regard. It quantified deformation, transient stress, and energy characteristics during drops or collisions and effectively characterized nonlinear and transient impact loads (Dimino et al., 2022). Through detailed mathematical models and computer simulations, it provided more accurate and consistent bruise prediction, especially for materials with complex internal structures or anisotropic properties.

Finite element analysis (FEA), as a numerical simulation method, simulated the mechanical behavior of complex structural materials under various loading conditions (Abbaszadeh et al., 2014). In recent years, FEA played a crucial role in heat transfer, vibration analysis, stress distribution, fatigue life prediction, and the study of the mechanical properties of composite materials (Van Zeebroeck et al., 2007). FEA was widely applied in bruise analysis of fresh agricultural products and in solving complex agricultural problems (Zulkifli et al., 2020). Kabas et al. (2008) used FEM to determine the deformation behavior of cherry tomatoes when they hit the ground after falling from a known height. Wu et al. (2013) investigated the pressure dispersion of bitter gourd under static pressure using FEM. According to the experimental results, the relative error in the static pressure bruise area of ​​bitter melon was close to 13 %. Ahmadi et al (2016a) explored variations in factors such as location and rate of fruits during collisions with other fruits and rigid bodies using FEM. Zhao et al (2019a) used 3D scanning and finite element simulations to assess collision bruising in goji berries. They employed the response surface method to determine the influence of drop height, collision material, and angle on bruising. However, the single isotropic homogeneous elastoplastic material models used in these studies were not suitable for materials with complex internal structures or anisotropic properties. The American ginseng main root had an irregular columnar shape, with distinct physical features between the cortex and cambium. Therefore, to investigate the dynamic response of the American ginseng main root under various collision conditions, a bilayer constitutive model was required.

This study aimed to analyze the bruise formation process and bruise resistance of the American ginseng main root under various collision conditions, with the following specific objectives: a) determine the physical features of the American ginseng cortex and cambium and establish an accurate bilayer constitutive model; b) validate the accuracy of the constitutive model by contrasting simulations with pendulum test results; c) conduct a numerical analysis of the dynamic mechanical response of the American ginseng main root during collisions; d) analyze the bruise resistance characteristics of the American ginseng main root under different conditions.

## 2 Materials and methods

### 2.1 Determination of physical features of American ginseng main root

#### 2.1.1 Density measurement

Ten American ginseng main roots with rootlets removed were selected as samples for the uniaxial compression test. The cortex and cambium of each American ginseng main root were separated using a cutting tool, and the density of each was measured separately. The density of the American ginseng cortex was measured using the specific gravity bottle method, while the density of the American ginseng cambium was determined using the specific gravity balance method (Xu et al., 2023). The density of the American ginseng cortex and cambium was calculated as shown in Equations 1, 2:


(1) $$\rho_{\text{s}}={\text{m}}_{\text{s}}\rho_{\text{w}}/({\text{m}}_{\text{sl}}-{\text{m}}_{\text{ol}})$$

(2) $$\rho_{\text{g}}=(m_{\text{bg}}-m_{\text{b}})\rho_{\text{w}}/[(m_{\text{w}}-m_{\text{b}})-(m_{\text{z}}-m_{\text{b}})]$$

where $\rho_{s}$ is the density of the American ginseng cambium (g cm^-^³), ${\text{m}}_{s}$ is the mass of the American ginseng cambium (g), ${\text{m}}_{\text{ol}}$ is the combined mass of the receptacle and the soaking liquid (g), ${\text{m}}_{\text{sl}}$ is the combined mass of the receptacle, soaking liquid, and the American ginseng cambium soaked in the liquid (g), $\rho_{\text{w}}$ is the density of water (g cm^-^³), $\rho_{\text{g}}$ is the density of the American ginseng cortex (g cm^-^³), ${\text{m}}_{\text{bg}}$ is the combined mass of the pycnometer and the American ginseng cortex (g), ${\text{m}}_{b}$ is the mass of the pycnometer (g), ${\text{m}}_{\text{w}}$ is the mass of the pycnometer filled with water (g), ${\text{m}}_{\text{z}}$ is the mass of the pycnometer filled with the American ginseng cortex and water (g).

#### 2.1.2 Physical features measurement

**Figure 1:**
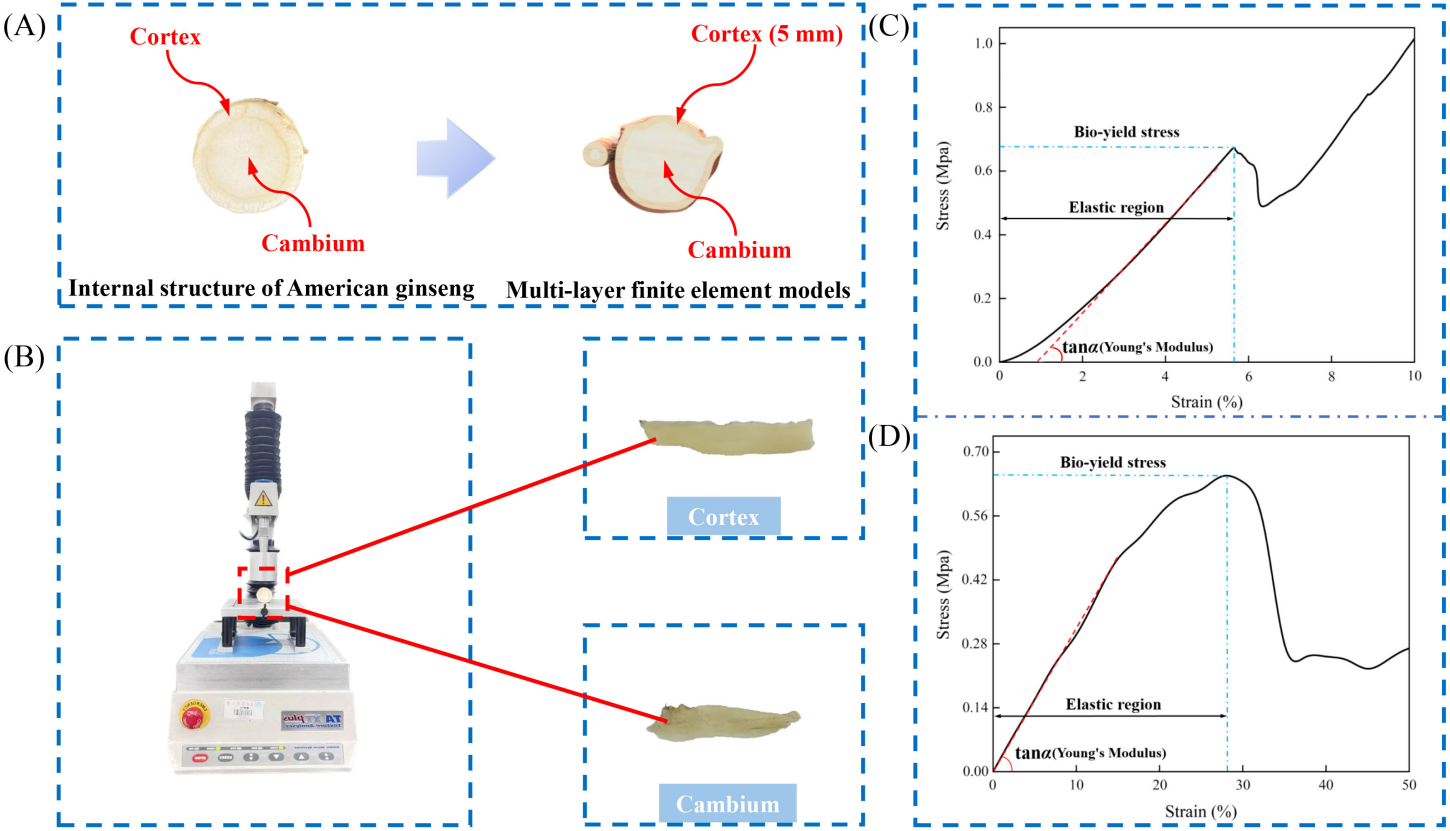
Measurement of physical features of American ginseng main root. **(A)** Internal structure of the American ginseng main root; **(B)** Uniaxial compression test of the American ginseng main root cortex and cambium; **(C)** Compression characteristics of the American ginseng main root cortex; **(D)** Compression characteristics of the American ginseng main root cambium.

Uniaxial compression tests on the cortex and cambium of the American ginseng main root were carried out using a texture analyzer (Stable Micro Systems, England) (**Figure 1B**). The contact force was set to 0.01 N, and the pressing speed to 10 mm min^-1^. Stress-strain curves for the cortex (**Figure 1C**) and cambium (**Figure 1D**) were generated from the experimental data. Stress in the cortex linear growth with strain up to the biological yield point, where the ratio of stress to strain represents the Young’s modulus (tanα) (Rashvand et al., 2022). After the yield point was exceeded, bruising occurred in the cortex. The cambium showed similar physical features. The procedure was performed 10 times, with the mean value calculated to ensure data accuracy. Poisson’s ratio was determined by calculating the ratio of lateral strain perpendicular to the load path to axial strain along the load path in the uniaxial compression test.

### 2.2 Constitutive model of American ginseng main root

**Figure 2:**
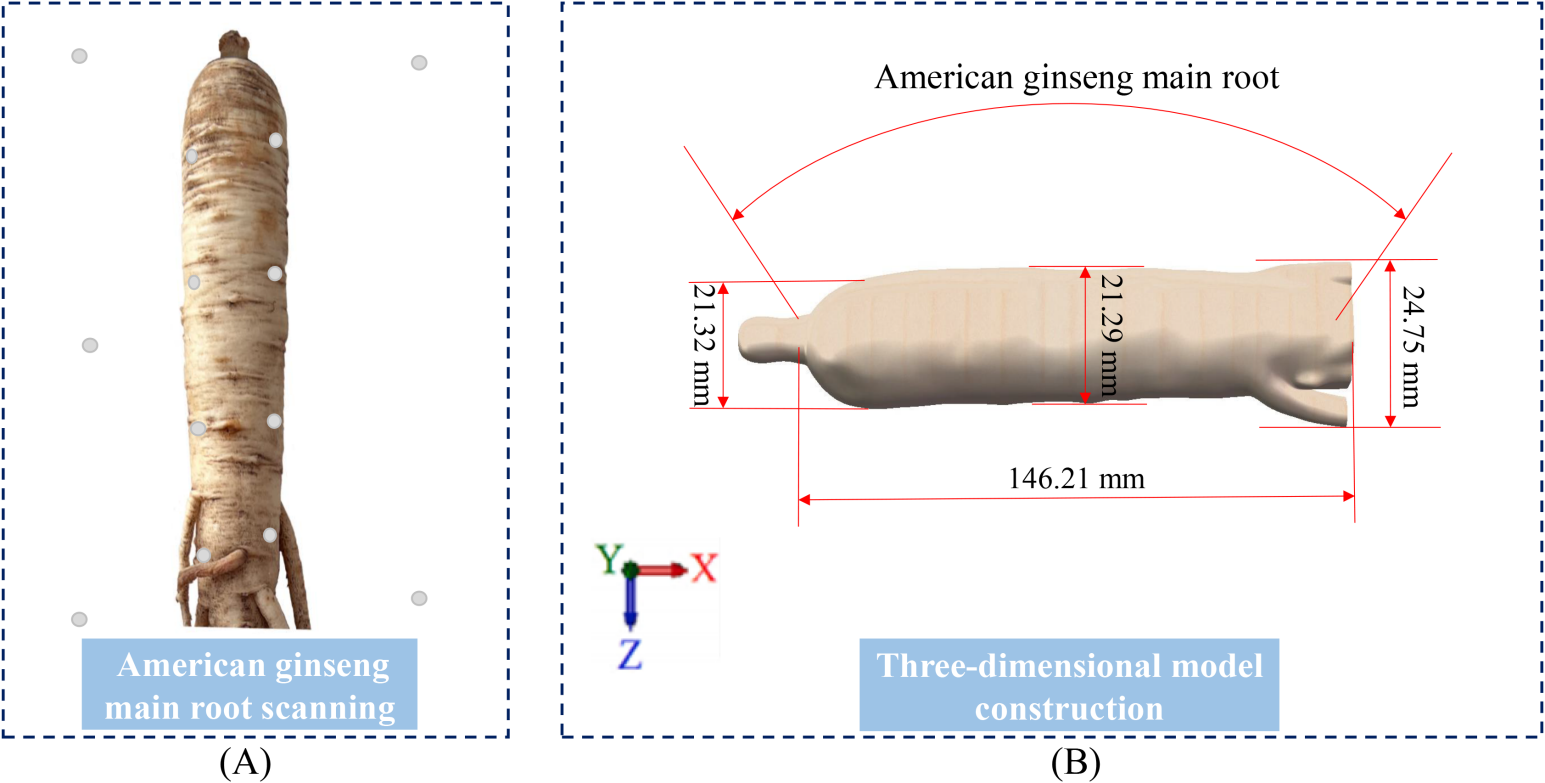
Reverse engineering of American ginseng main root. **(A)** Scanning of the American ginseng main root; **(B)** Solid 3D modelling in SolidWorks.

This study employed reverse engineering to create a solid model of the American ginseng main root (Guan et al., 2023). A handheld intelligent laser 3-dimensional scanning device (MarvelScan handheld intelligent laser 3D scanner, Shenzhen SR measurement technology Co., Ltd., Shenzhen, China) was employed to collect point cloud data of the American ginseng main root (**Figure 2A**). After merging the point cloud data, surface splicing was carried out using Geomagic Studio (Geomagic Inc., Morrisville, USA). The American ginseng main root was then solid modelled in SolidWorks 2022 (Dassault Systemes S. A, Massachusetts, USA) to verify the geometric parameters of its outer outline (**Figure 2B**). Following the application of the techniques—point cloud splicing, surface splicing, and model reconstruction—a three-dimensional solid model comprising two distinct components, the cortex (5 mm) and the cambium (**Figure 1A**), was ultimately constructed. The cambium was then connected to the cortex through surface contact.

The bilayer constitutive model of the American ginseng main root presented in this study is based on its physical and mechanical properties. In the quasi-static compression test, the American ginseng cambium primarily exhibits elastic deformation, leading to its classification as an isotropic linear elastic material model. The American ginseng cortex was observed to possess high toughness and some degree of plasticity, allowing for the application of an idealized bilinear isotropic strain-hardening elastic-plastic model.

### 2.3 Experimental content and methods

#### 2.3.1 Pendulum test

**Figure 3:**
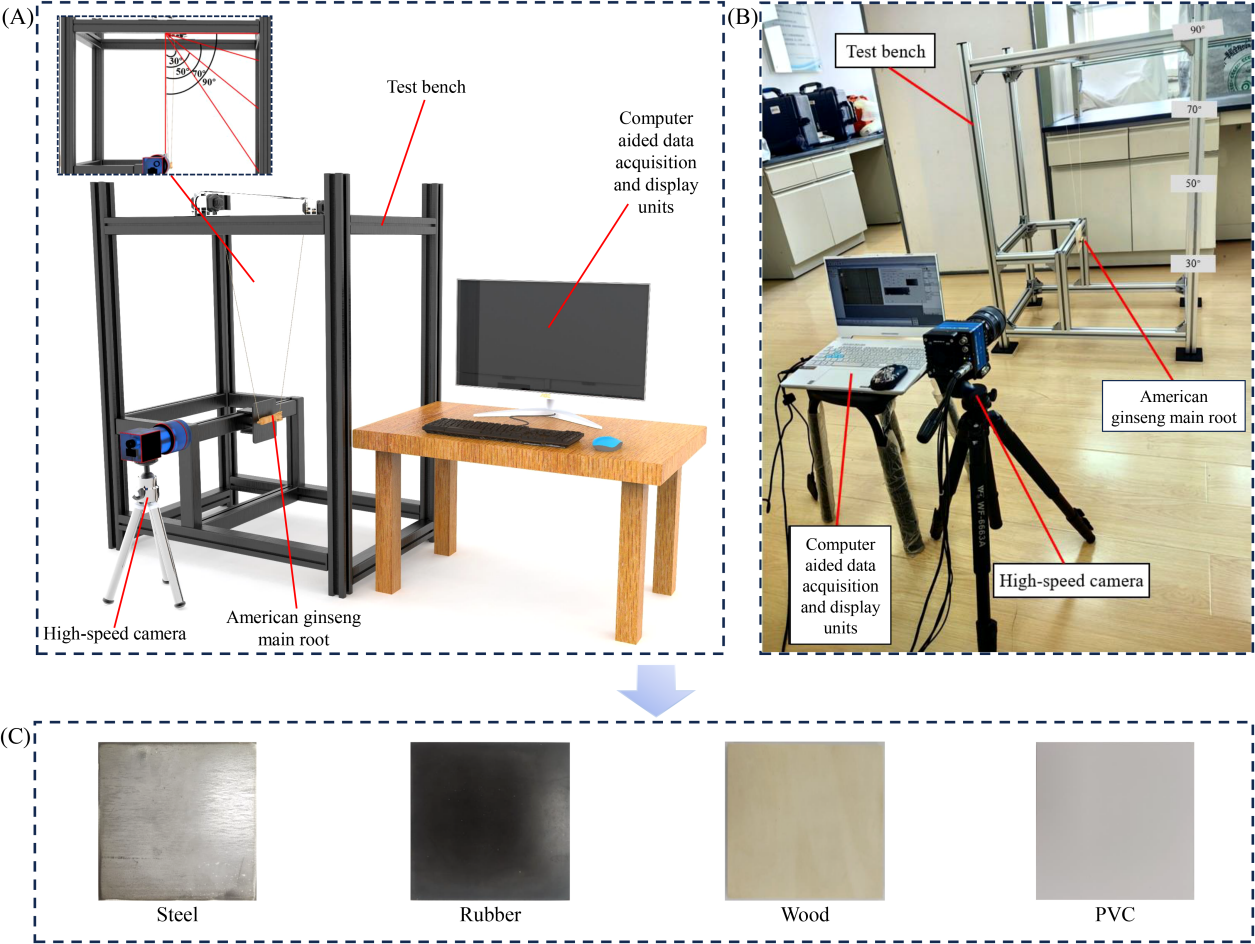
Pendulum test and contact materials. **(A)** Pendulum test apparatus; **(B)** Experimental environment; **(C)** Contact materials used in the experiment.

A pendulum test apparatus was designed for collision testing of American ginseng main roots (**Figure 3A**). The ends of the American ginseng main root sample were attached to the crossbeam at the top of an aluminum profile frame using cotton threads. The American ginseng main root was raised to specific drop angles (30°, 50°, 70°, and 90°) and then allowed to fall freely, striking the square plate installed on the frame. The plate materials were steel, rubber, wood, and PVC (**Figure 3C**). A high-speed camera (Phantom Multicam, Phantom Video Player, PCC 2.8) mounted on a tripod recorded the collision at 2000 fps and acquired images at a resolution of 1296 × 1024 pixels (**Figure 3B**). The camera captured the moment of contact during the collision of the American ginseng main root samples and recorded the velocity of the samples before and after the collision. Each drop angle test was repeated ten times.

#### 2.3.2 Finite element simulation of American ginseng pendulum collision

The LS-DYNA unit in ANSYS 2022 (ANSYS, Inc., Pennsylvania, USA) was used for simulations, including 16 different scenarios that considered various materials in contact with the American ginseng main root (steel, rubber, wood, and PVC) and drop angles of 30°, 50°, 70°, and 90°. Considering the deformation process following energy absorption from the collision and the contact rebound phase, the solution time for each scenario was fixed at 0.02 s.

### 2.4 Simulation model validation test

**Figure 4:**
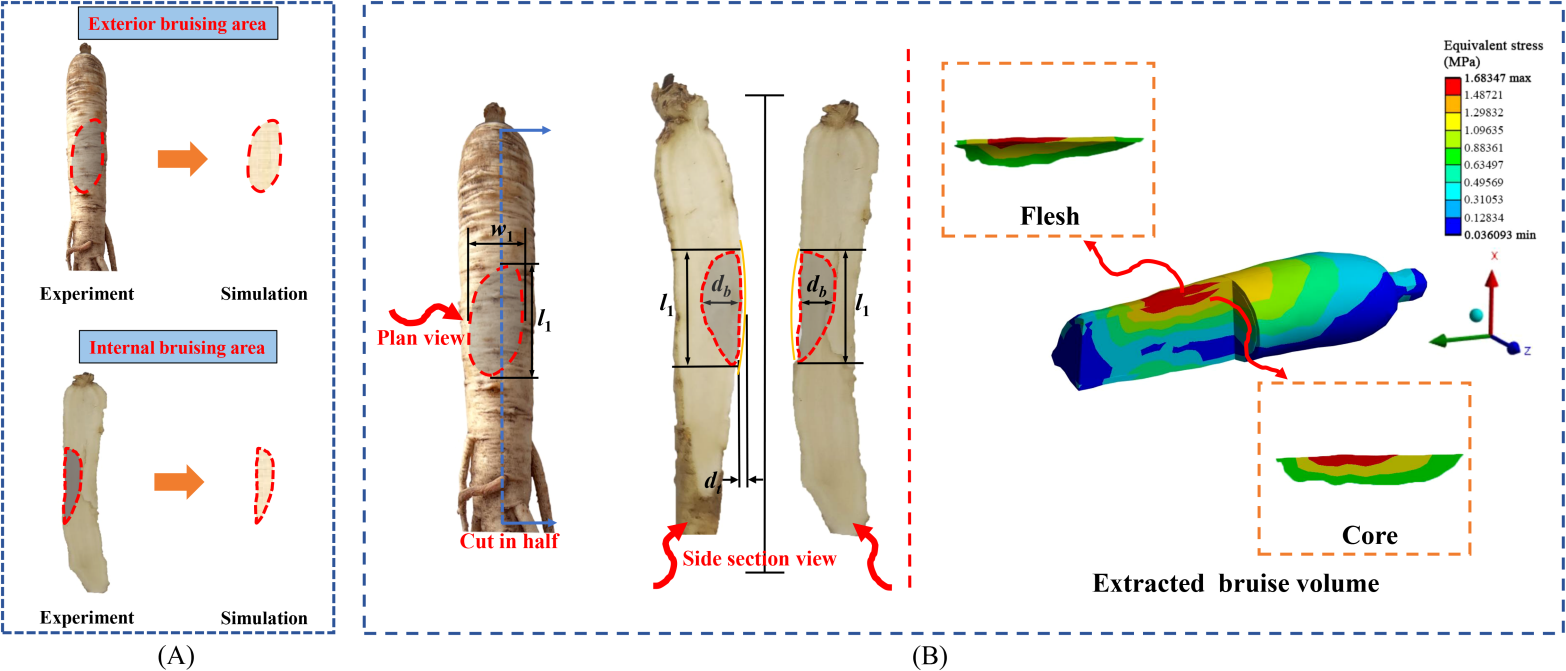
Extraction of bruise area and bruise volume of American ginseng main root. **(A)** Comparison of exterior and internal bruise areas in simulation and pendulum test; **(B)** Process of bruise volume extraction.


This study utilized the pendulum test to validate the bruising sustained by American ginseng during transportation and harvesting. The entire collision process was captured using a high-speed camera, and the images were processed to analyze the contours of bruised areas on the exterior of the American ginseng main root (**Figure 4**). By analyzing the velocity changes in the simulation, Equation 3 was employed to calculate the drop velocity of the American ginseng main root in the test:

(3) $$v_{e}=(s_{e+1}-s_{e})/(t_{e+1}-t_{e})$$

where $v_{e}$ is the velocity at time *e* (mm s^-1^), $t_{e+1}$ and $t_{e}$ are the time points at which the high-speed camera records the falling state of the American ginseng main root (s), $s_{e+1}$ and $s_{e}$ are the displacements at times e+1 and e (mm).

The relative error calculation formula was employed to determine the velocity difference between the pendulum test and the simulation, thereby validating the accuracy of the established constitutive model, as shown in Equation 4:


(4) $$\sigma_{e}=(v_{e}-v_{es})/v_{e}\times 100\sum$$

where $\sigma_{e}$ is the velocity error at time e (%), and $v_{es}$ is the velocity at time *e* in the simulation (mm s^-1^).

A fruit hardness tester (Zhejiang Top Instrument Co., Ltd., Hangzhou, China) was used to measure the hardness of the American ginseng main root before and after the collision, enabling the calculation of the total bruised volume. The bruised volume of the American ginseng main root was calculated using Equation 5:


(5) $$BV=\pi (d_{b}-d_{t})(3l_{1}w_{1}+4{\left(d_{b}-d_{t}\right)}^{2})/24$$

where $l_{1}$ and $w_{1}$ are the length and width of the bruised area on the exterior of the American ginseng main root (mm), $d_{b}$ is the bruise deepness of the American ginseng main root after the pendulum test (mm), $d_{t}$ is the distance from the epidermis to the top of the bruise (mm), and BV is the bruise volume of the American ginseng main root (mm^3^).

In the pendulum test, the energy of the American ginseng main root after the collision was derived from its potential energy:


(6) $$E_{\text{i}}=mgH_{\text{d}}=mgl\text{ (}1-cos\alpha )$$

where $E_{i}$ is the energy generated by the American ginseng main root after the collision (mJ), *m* is the mass (g), $H_{\text{d}}$ is the drop height (mm), *l* is the length of the string (mm), α is the original swing angle (°), and *g* is the gravitational acceleration (mm s^-2^).

Following the collision, the American ginseng main root rebounded to its maximum height. The difference between the original and final potential energies, which is absorbed during the collision, was expressed as:


(7) $$E_{a}=mg(H_{\text{d}}-H_{\text{r}})=mgl\text{ (}cos\beta -cos\alpha )$$

where $E_{a}$ is the energy absorbed by the American ginseng main root (mJ), $H_{\text{r}}$ is the maximum rebound height (mm), β is the rebound angle at the top (°).

By combining Equations 6, 7, the total energy during the American ginseng collision process was obtained as:


(8) $$E=E_{i}+E_{a}$$

The total energy during the collision of the American ginseng main root (excluding friction and contact energy) was calculated using Equation 8. A vernier calliper (manufactured by Shandong Greener Precision Instruments Co., Ltd., with a precision of 0.01mm) was used to measure the width, length, and depth of the American ginseng main root bruise. The bruise resistance index ( BRI ) of the American ginseng main root was then calculated using the bruise volume and the total energy absorbed in the collision.

BRI was the ratio of the load parameters endured by agricultural products during dynamic collision to the amount of bruised tissue resulting from these parameters (Gołacki et al., 2009), as shown in Equation 9:


(9) BRI=E/BV

where *E* is the total energy absorbed by the American ginseng main root during the drop (mJ), BV is the bruise volume of the American ginseng main root after the collision (mm^3^), and BRI is the bruise resistance index of the American ginseng main root (mJ mm^-3^).

### 2.5 Calculation of bruise area and extraction of bruise volume

The bruise area of the American ginseng main root was calculated, followed by a quantitative comparison between the test and simulation results. The comparison encompassed the exterior bruise area and the internal bruise area, with the latter obtained by cutting along the centerline of the bruise surface (**Figure 4A**). The long axis dimensions of the bruised area in the pendulum test were determined using a vernier caliper, and photos of the bruised surface were uploaded to CAXA software (version: 2016, Beijing Digital Dafang Technology Co., Ltd., Beijing, China) to acquire the long axis dimensions and calculate the bruise area. In the simulation, the long axis dimensions of the bruised area were determined using the LS-DYNA unit and then imported into CAXA to calculate the bruise area.

After the collision finite element simulation, the maximum stress was contrasted with the yield stress of the American ginseng cortex and cambium. The results indicated that bruising occurred in regions where the stress surpassed the yield stress. The combined bruised volume of the cortex and cambium constituted the total bruised volume of the American ginseng main root. The bruised portion was extracted from simulation software and saved in STL format. The STL format was then uploaded to SolidWorks and transformed into a STEP format for visualization of the bruised volume (**Figure 4B**).

## 3 Results and discussion

### 3.1 Results of physical feature tests

The material features of the American ginseng cortex and cambium were obtained through uniaxial compression tests. Additionally, the material features of steel, wood, rubber, and PVC were sourced from the ANSYS material library (ANSYS 2022 R1) (**Table 1**).

**Table 1:** Parameters of American ginseng main root and contact materials.

Materials	Properties	Values
American ginseng main root cortex	Poisson’s ratio	0.42
	Density (g cm^-3^)	1.16
	Young’s modulus (MPa)	12.11
	Yield strength (MPa)	0.68
American ginseng main root cambium	Poisson’s ratio	0.35
	Density (g cm^-3^)	0.95
	Young’s modulus (MPa)	2.32
	Yield strength (MPa)	0.65
Steel	Poisson’s ratio	0.30
	Density (g cm^-3^)	7.85
	Young’s modulus (MPa)	200000
Rubber	Poisson’s ratio	0.47
	Density (g cm^-3^)	1.2
	Young’s modulus (MPa)	–
Wood	Poisson’s ratio	0.38
	Density (g cm^-3^)	0.7
	Young’s modulus (MPa)	–
PVC	Poisson’s ratio	0.4
	Density (g cm^-3^)	1.39
	Young’s modulus (MPa)	2861

### 3.2 Mesh sensitivity analysis

**Figure 5:**
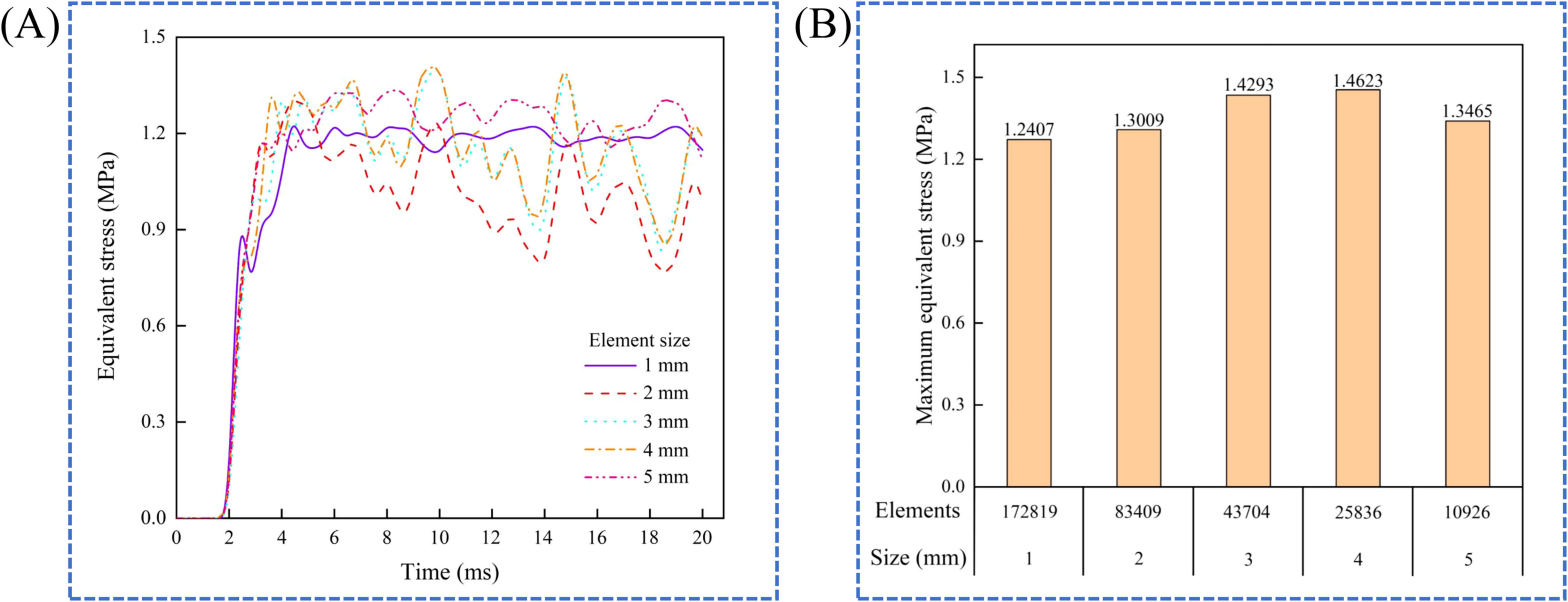
Mesh sensitivity analysis. **(A)** Equivalent stress for different element sizes; **(B)** Maximum equivalent stress for different elements counts.

This study determined the appropriate element size through mesh sensitivity analysis. The model sequentially used element sizes ranging from 1 mm to 5 mm for collision simulations. The smallest element size that adequately represented the American ginseng main root was selected based on the results of the mesh sensitivity analysis and acceptable computational time. The curves of equivalent stress over time for different element sizes were generated (**Figure 5A**). The maximum equivalent stress was reached with an element size of 4 mm (**Figure 5B**). Thus, an element size of 4 mm was best suited to capture equivalent stress. The constitutive model of the American ginseng main root in the simulation consisted of 25836 elements with a 4 mm element size.

### 3.3 Model verification and simulation

#### 3.3.1 Model verification

**Figure 6:**
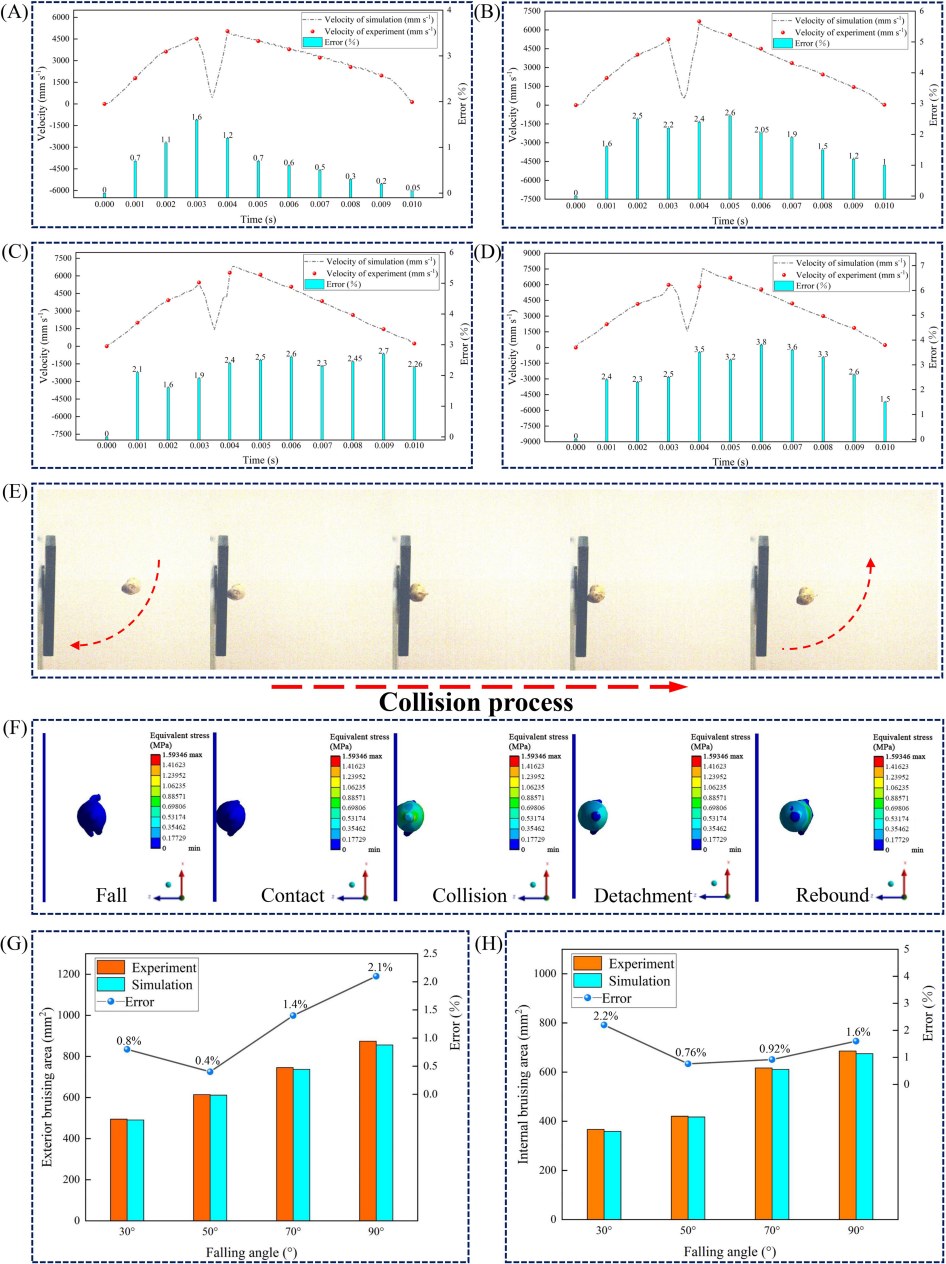
Comparison of collision processes and characterization of bruise area. **(A–D)** Velocity changes of the American ginseng main root with steel as the contact material at drop angles of 30°, 50°, 70°, and 90°, respectively; **(E)** Drop process captured by high-speed imaging test; **(F)** Finite element simulation of the American ginseng main root collision process; **(G)** Exterior bruise area and error from simulation and pendulum tests at different drop angles; **(H)** Internal bruise area and error from simulation and pendulum tests at different drop angles.

The collision process of the American ginseng main root can be divided into five stages: fall, contact, collision, detachment, and rebound (**Figures 6E, F**). The accuracy of the constitutive model was confirmed by comparing velocity changes and bruise areas during the collision process.

Velocity-time curves during the collision process were plotted (**Figures 6A–D**). Velocity gradually increased during the fall and decreased after contact. During the rebound process, the velocity reached its peak value and then gradually declined. The greater the drop angle, the higher the rebound velocity. In the solution process, the nonlinear behavior of materials or contact conditions was not fully or accurately reflected in the model, potentially causing differences between the simulation results and pendulum test results (Yan et al., 2024). A comparison between the simulation and pendulum test results indicated that the highest velocity error during the collision process was 3.8 % (**Figure 6D**).

Five sets of tests were conducted for each drop angle (with steel as the contact material), and the exterior and internal bruise areas of the American ginseng main root were measured separately, with the mean values calculated. Additionally, the error between the simulation and the pendulum test was calculated (**Figures 6G–H**).

At a drop angle of 50°, the exterior bruise area of the American ginseng main root was relatively small, measuring 611.74 mm² (simulation) and 614.26 mm² (pendulum test), with the smallest error of 0.4 %. At a drop angle of 90°, the exterior bruise area of the American ginseng main root reached its maximum, measuring 855.74 mm² (simulation) and 874.13 mm² (pendulum test), with the largest error of 2.1 %.

At a drop angle of 30°, the internal bruise area of the American ginseng main root was the smallest, measuring 359.17 mm² (simulation) and 367.21 mm² (pendulum test), with the largest error being 2.2 %. At a drop angle of 50°, the error in the internal bruise area of the American ginseng main root was the smallest, at 0.76 %. At a drop angle of 90°, the internal bruise area of the American ginseng main root reached its maximum, measuring 675.57 mm^2^ (simulation) and 686.52 mm^2^ (pendulum test).

Within the allowable error range, the comparison of the American ginseng main root velocity, exterior bruise area, and internal bruise area between the simulation and pendulum test demonstrated the accuracy of the constructed constitutive model of the American ginseng main root.

#### 3.3.2 Finite element simulation

**Figure 7:**
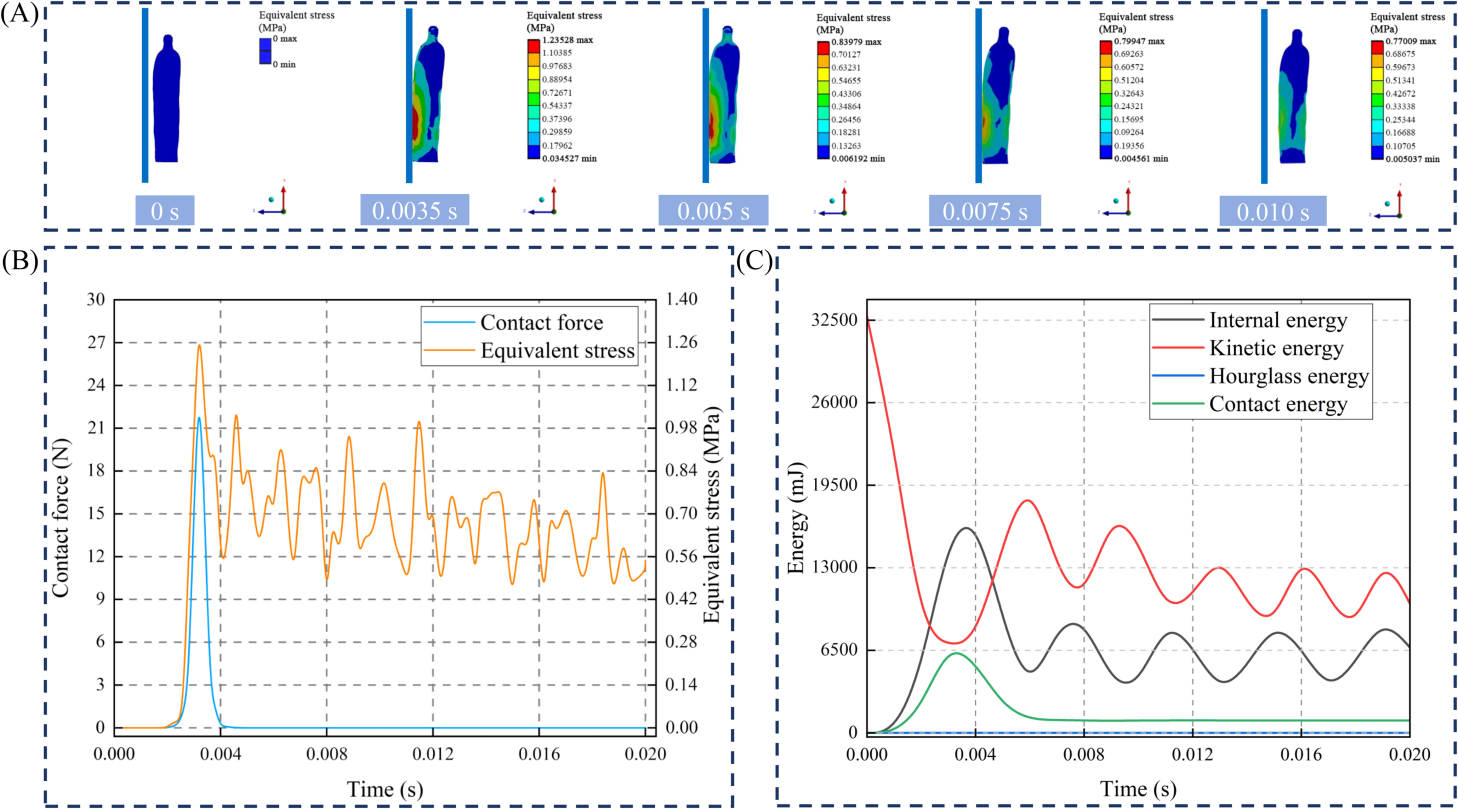
Forces and energy during American ginseng main root collision. **(A)** Finite element simulation of American ginseng main root collision; **(B)** Time-dependent curves of equivalent stress and contact force during the collision; **(C)** Curves of the four types of energy variations during the collision.

The collision process of the American ginseng main root at a 90° drop angle with steel as the contact material was analyzed. Following the collision, the contact force and equivalent stress in the American ginseng main root gradually increased (**Figure 7A**). At 3.5 × 10^-3^ s, the equivalent stress and contact force in the collision area of the American ginseng main root reached maximum values of 1.235 MPa and 22.152 N, respectively (**Figure 7B**). The equivalent stress in the non-collision area of the American ginseng main root was minimal, recorded at 4.561 × 10^−3^MPa. After rebounding, the American ginseng main root gradually detached from the contact material, causing the contact force to sharply drop to zero. Due to the large equivalent stress generated during the collision, it did not dissipate immediately. Instead, the equivalent stress propagated within the American ginseng main root, exhibiting periodic fluctuations that gradually decay to zero.

The variations in four forms of energy during the American ginseng main root collision process were analyzed (**Figure 7C**). During the collision of the American ginseng main root, energy was absorbed or transformed, causing the kinetic energy to rapidly decrease from its maximum value of 32500 mJ to a minimum. Simultaneously, internal energy increased to a maximum of 16252 mJ. Subsequently, both kinetic energy and internal energy exhibited periodic fluctuations, possibly due to energy changes resulting from elastic or plastic deformation (Liu et al., 2024).

### 3.4 Dynamic mechanical response of American ginseng main root at different drop angles

**Figure 8:**
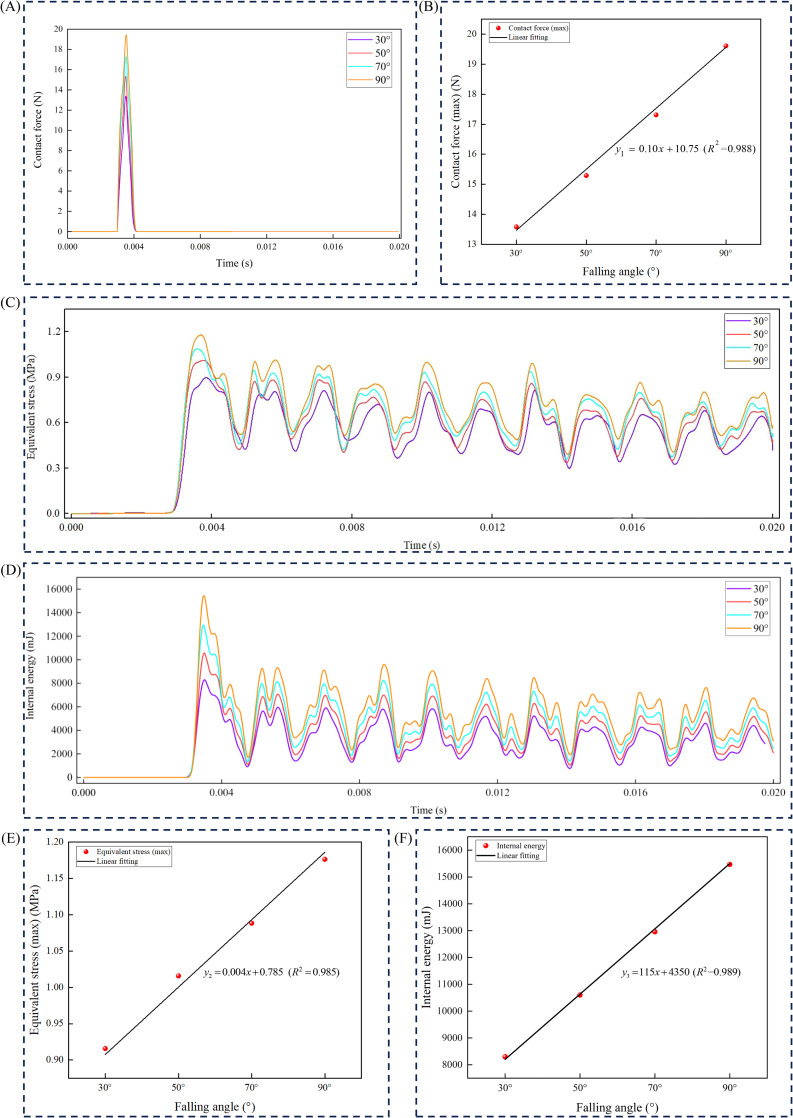
Relationship between maximum contact force, maximum equivalent stress, and maximum internal energy with drop angle. **(A)** Curve of maximum contact force as time progresses; **(B)** Relationship between maximum contact force (*y*_1_) and different drop angles (*x*); **(C)** Curve of maximum equivalent stress as time progresses; **(D)** Curve of maximum internal energy as time progresses; **(E)** Relationship between maximum equivalent stress (*y*_2_) and different drop angles (*x*); **(F)** Relationship between maximum internal energy (*y*_3_) and different drop angles (*x*).

Drop angles of 30°, 50°, 70°, and 90° were used as test conditions, with wood as the material. The maximum contact force, maximum equivalent stress, and maximum internal energy were analyzed over time (**Figure 8**). When the American ginseng main root contacted the wooden board, the contact force instantly reached its peak value. As time progressed, the contact force gradually decreased until the American ginseng main root detached from the contact material, at which point the contact force dropped to its minimum (**Figure 8A**). Linear fitting results indicated that the maximum contact force of the American ginseng main root was positively correlated with the drop angle (**Figure 8B**). The greater the drop angle, the higher the peak of the maximum contact force. At a drop angle of 90°, the maximum contact force was 19.61 N, while at a drop angle of 30°, it was 13.58 N. The maximum equivalent stress peaked at 3.5 × 10^−3^s and gradually decreased over time. Due to residual stress after the collision, the equivalent stress exhibited periodic fluctuations (**Figure 8C**). At a drop angle of 90°, the maximum equivalent stress was 1.176 MPa, while at a drop angle of 30°, it was 0.916 MPa. Linear fitting results indicated that the maximum equivalent stress of the American ginseng main root was positively correlated with the drop angle (**Figure 8E**). The greater the drop angle, the higher the peak of the maximum equivalent stress. The variations in the maximum internal energy of the American ginseng main root over time at various drop angles were analyzed (**Figure 8D**). During a collision, the maximum internal energy rapidly increased, peaking at 3.5 × 10^−3^s, and then gradually decreased with periodic fluctuations. With the increase in drop angles, the peak value of the maximum internal energy also increased. At a drop angle of 90°, the maximum internal energy was 15468 mJ, while at a drop angle of 30°, it was 8293 mJ. Linear fitting results indicated that the maximum internal energy was positively correlated with the drop angle (**Figure 8F**).

### 3.5 Dynamic mechanical response of American ginseng main root under different contact materials

**Figure 9:**
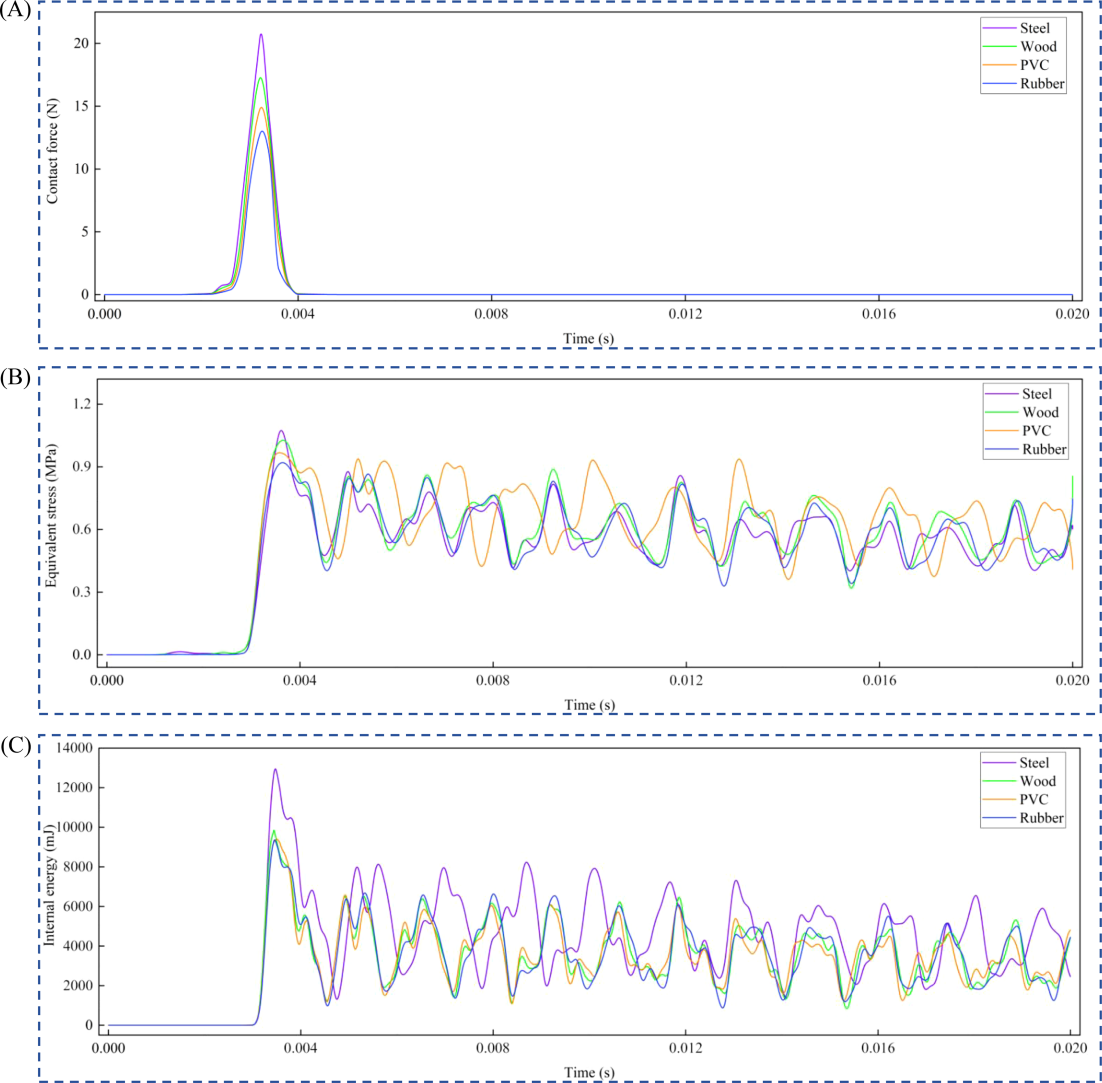
Variation of maximum contact force, maximum equivalent stress and maximum internal energy with time for different contact materials. **(A)** Curve of maximum contact force as time progresses; **(B)** Curve of maximum equivalent stress as time progresses; **(C)** Curve of maximum internal energy as time progresses.

The materials used were steel, wood, PVC, and rubber at a drop angle of 70°. The maximum contact force, maximum equivalent stress, and maximum internal energy were analyzed over time (**Figures 9A–C**). The maximum contact force was highest when the contact material was steel, at 20.215 N, and lowest when the contact material was rubber, at 12.613 N (**Figure 9A**). The maximum equivalent stress was highest when the American ginseng main root was in contact with steel, at 1.183 MPa, and lowest at 0.928 MPa when in contact with rubber (**Figure 9B**). As the hardness of the contact material higher, the maximum equivalent stress and maximum contact force generated during the collision of the American ginseng main root also increased, leading to greater bruising. Additionally, the maximum internal energy was highest when the contact material was steel, at 12959 mJ, and lowest when the contact material was rubber, at 8570.5 mJ (**Figure 9C**).

### 3.6 Bruise resistance characteristics of American ginseng main root

**Figure 10:**
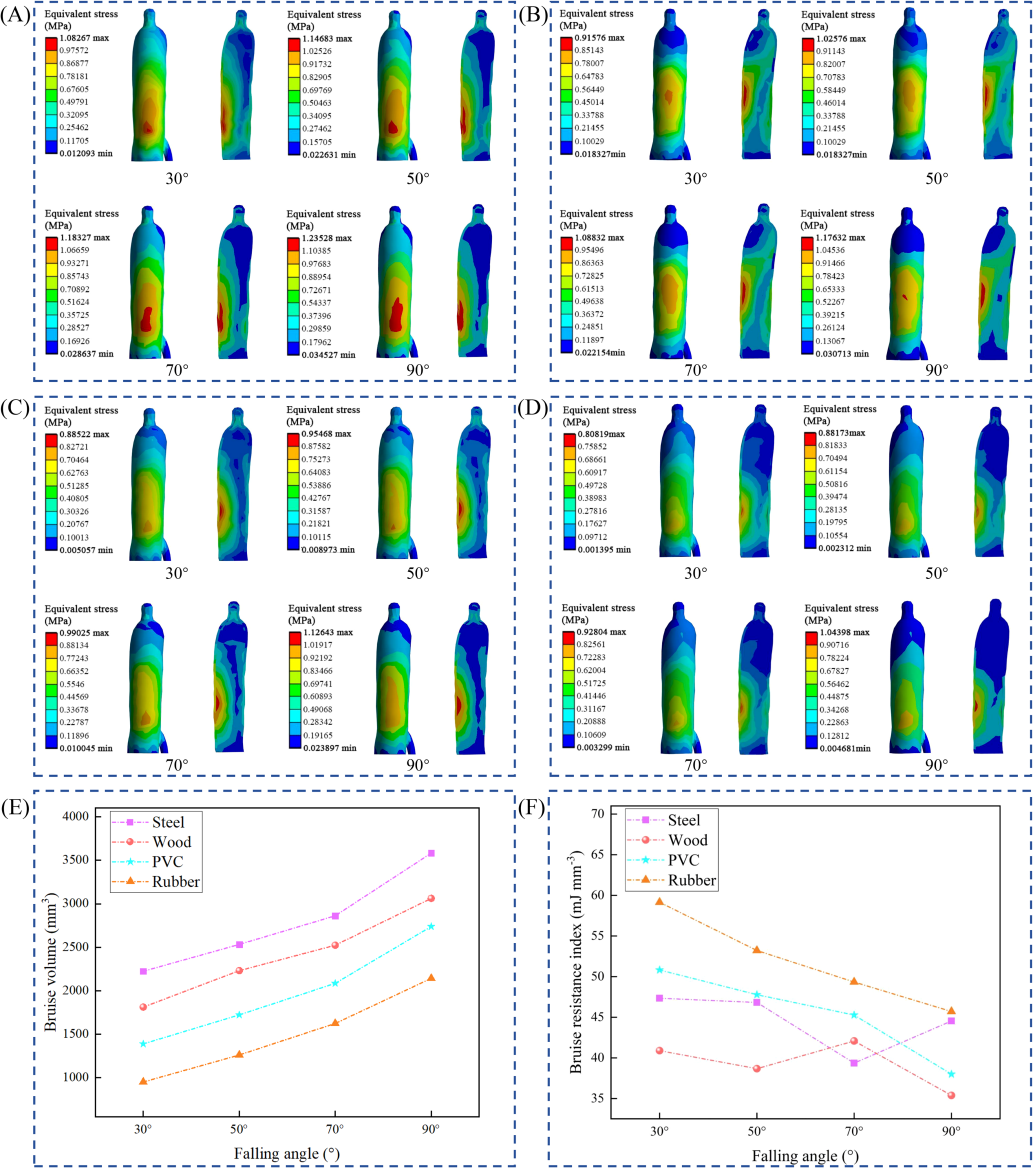
Bruise resistance characteristics of American ginseng main root. **(A–D)** Equivalent stress distribution cloud maps of the American ginseng main root with contact materials of steel, wood, PVC, and rubber at drop angles of 30°, 50°, 70°, and 90°; **(E, F)** Bruise resistance index and bruise volume of different contact materials as the drop angle varies.

Stress cloud maps of the American ginseng main root at different drop angles and with different contact materials were extracted (**Figures 10A–D**). The stress distribution in the American ginseng main root was uneven, with stress values gradually decreasing from the center toward the periphery. Stress values in the bruised areas were higher than those in the non-bruised areas; in other words, bruising only occurred where the stress was sufficiently high. This stress distribution pattern led to bruising in specific areas of the American ginseng main root. Therefore, assessing the bruise resistance characteristics of agricultural products requires quantifying the extent of bruise in these specific areas.

The bruise resistance index reflects the amount of bruise per unit of energy induced in crops during a dynamic collision and can be used to assess the degree of bruise in rhizome crops. Bruise volumes under different materials (steel, wood, PVC, and rubber) were statistically analyzed as the drop angle (30°, 50°, 70°, and 90°) varied (**Figure 10E**). For steel and wood, bruise volume significantly increased with the drop angle, with the maximum bruise volume being 3583.26 mm^3^ and 3062.19 mm^3^, respectively. The bruise volume with PVC as the material also increased with the drop angle, but the overall trend is lower than that of steel and wood, with the smallest bruise volume of 1392.41 mm^3^ at a drop angle of 30° and the largest bruise volume of 2739.37 mm^3^ at a drop angle of 90°. The simulation results indicated that the bruise volume caused to the American ginseng main root by different materials, from largest to smallest, was steel > wood > PVC > rubber. Rubber materials are more effective in reducing bruising to the American ginseng main root. For example, at a drop angle of 30°, the bruise resistance index of the American ginseng main root was 59.14 mJ mm^-3^ with rubber and 40.89 mJ mm^-3^ with wood. At a drop angle of 90°, the bruise resistance index was 45.72 mJ mm^-3^ with rubber and 35.38 mJ mm^-3^ with wood. At the same drop angle, the American ginseng main root bruise resistance index was higher with rubber as the contact material than with the other three materials (**Figure 10F**).

The bruise resistance of the American ginseng main root gradually decreased as the drop angle increased. As the drop angle increased, the contact surface with the material expanded. At larger drop angles, the central tissue of the American ginseng main root endured greater contact forces. According to the pattern where stress is maximized close to the contact surface and progressively diffuses interior, the American ginseng main root exhibited characteristics of localized bruising.

### 3.7 Discussion

In most finite element simulation studies, the multilayer structural properties of rhizome crops are often overlooked. Typically, agricultural products are simplified to a unitary structure (Caglayan et al., 2018; Celik, 2017). This simplification is suitable for some crops with analogous overall mechanical features, but for American ginseng, it could affect the accuracy of the results. Studies have shown that the multilayer structure of rhizome crops significantly impacts their physical and mechanical properties. For example, Stopa et al. (2019) found in their study on carrots that different tissue layers exhibited distinct mechanical behaviors when subjected to force. Li et al. (2013) also pointed out that overlooking the multilayer structure of crops could lead to inaccurate estimates of their fracture and deformation behaviors. This is especially true for complex rhizome crops like American ginseng, which have varying physical characteristics within their internal structures. This study applied a reverse engineering method to establish constitutive model of the American ginseng main root besides conducted precise physical parameter measurements. This approach not only improved the accuracy of the simulations but also provided a reference for future research. The American ginseng main root was divided into the cortex and cambium for separate mechanical property testing. This method partially overcame the limitations of previous studies that simplified rhizome crops to a single tissue, more accurately reflecting the actual conditions of collision bruising.

Since some studies involve practical calculation methods as well as the validation and comparison of models in simulations (Stropek and Gołacki, 2020), selecting an appropriate analysis method for model validation is crucial. Currently, many studies primarily use finite element simulation to evaluate the bruise extent of agricultural products (Ahmadi et al., 2016a), indicating that the FEM is a reliable and effective means for evaluating collision bruising in agricultural products. This study, based on finite element simulation and high-speed imaging technology, validated the drop velocity and bruise area of the American ginseng main root under different collision conditions, achieving the highest velocity error of 3.8 %. This result is consistent with the falling process and bruising observed during the pendulum test, indicating that the intrinsic model established in this study has a high degree of accuracy. This model calibration method provides a reliable verification approach for subsequent studies. Future studies could consider a more diverse range of fruit and vegetable varieties, as well as more complex collision scenarios, including multiple collisions, multi-object collisions, and collisions with different composite material surfaces. Additionally, new parameters, such as humidity, temperature, and maturity, could be introduced to further enhance model adaptability and predictive capability.

The observed crossover of bruise resistance index (BRI) curves for different contact materials at specific drop angles likely arises from inherent differences in the mechanical properties and energy transfer mechanisms of the materials. Notably, a nonlinear relationship among hardness, elastic modulus, and energy absorption characteristics exists (Zhao et al., 2019a). Hard materials typically exhibit higher elastic moduli, yet they tend to absorb less energy during impact, leading to a rapid transfer of the majority of energy to the impacted object. Conversely, softer materials, characterized by a lower elastic modulus, often provide superior energy absorption capabilities, thereby mitigating damage under certain conditions (Ahmadi et al., 2016a). Consequently, at specific drop angles, hard materials may manifest a higher BRI, whereas at other angles, the enhanced energy absorption of softer materials may yield a higher BRI. This phenomenon underscores the necessity of considering multiple mechanical properties in the design of impact-mitigation systems and the evaluation of collision safety, rather than relying solely on a single parameter. This study analyzed the dynamic mechanical response of the American ginseng main root under different drop angles and collision contact conditions, resulting in variation curves of the maximum contact force, maximum equivalent stress, and maximum internal energy during the collision process (**Figures 8**, **9**). Analysis of the forces during the American ginseng main root collision showed that the maximum contact force and maximum equivalent stress were primarily concentrated near the contact surface and gradually diffused inward. This stress distribution pattern determined the shape and extent of the bruised area. Although the problem of bruising from collisions of agricultural products can be effectively analyzed using sophisticated near-infrared spectroscopy and computed tomography technologies, these methods require a high technological threshold and cost. When carrying out spot checks on agricultural product specimens or assessing the functionality of equipment, using bruise volume and surface area to calculate the extent of bruising is unquestionably the most straightforward and effective method. In this research, the bruise volume of the American ginseng main root under different collision conditions was compared. The results indicated that as the drop angle enlarged, the bruise volume of the American ginseng main root also increased, leading to the higher risk of bruising. Finite element simulation results revealed that the bruise volume of the American ginseng main root varied significantly when colliding at different drop angles and with different contact materials such as steel, wood, PVC, and rubber. Under conditions of larger drop angles and harder contact materials (such as steel and wood), the American ginseng main root exhibited greater bruising, with maximum bruise volumes of 3583.26 mm^3^ and 3062.19 mm^3^, respectively. Although the developed model and experimental setup provided valuable insights, several limitations must be acknowledged. Firstly, the constitutive model assumed linear elasticity for the cambium and a simplified elastic–plastic behavior for the cortex, whereas actual plant tissues exhibit viscoelasticity and are strain-rate sensitive. Thus, under very high strain rates or over longer durations post-impact, the material response may diverge from the predictions of the current model. Incorporating viscoelastic properties in future models would improve accuracy across a range of loading conditions. Second, the model and experiments focused solely on single-impact events. In practice, during harvest or transport, American ginseng main roots may undergo multiple successive impacts or continuous vibrations; however, the current model does not account for cumulative bruise from repeated impacts. Third, the pendulum test simulates a free-swing impact scenario that may not capture all aspects of actual harvester impacts, such as interactions with soil or adjacent roots; extending validation to field conditions would further strengthen the conclusions. Fourth, individual variability in American ginseng root morphology and moisture content was not explicitly modeled, as average properties were used—although actual bruising may vary significantly among American ginseng main roots. Finally, high-speed photography in this study was primarily used for velocity measurement and bruise imaging; more advanced imaging techniques, such as X-ray CT scanning for internal bruises, could provide a more detailed validation of internal damage patterns. Addressing these limitations in future work—for example, by incorporating viscoelastic FEM models, simulating repeated impacts, and testing a broader range of crop samples—will enhance both the robustness and applicability of the findings.

This study primarily focused on the American ginseng main root, but the methods could also be applied to other rhizome crops. The application of these methods could be considered in subsequent studies to enhance the understanding and assessment of collision bruising in various rhizome crops. This study provides a theoretical basis for designing scientifically sound American ginseng harvesting machinery, helping to reduce bruising during harvesting and improve both yield and quality. It also serves as a reference for designing mechanized harvesting equipment for other rhizome crops. Future research can build on this foundation by further optimizing model parameters and exploring bruising mechanisms under a wider range of conditions, providing more comprehensive technical support for the fields of agricultural machinery and food engineering.

## 4 Conclusion

1. The bruise volume increased with the drop angle, and the maximum bruise volume was 3583.26 mm^3^ (steel) and 3062.19 mm^3^ (wood), respectively. At a drop angle of 30°, the bruise resistance indices of the American ginseng main root were 59.14 mJ mm^-3^ (rubber) and 40.89 mJ mm^-3^ (wood). At a drop angle of 90°, the bruise resistance indices of American ginseng main root were 45.72 mJ mm^-3^ (rubber) and 35.38 mJ mm^-3^ (wood).
2. The maximum equivalent stress, maximum contact force, and maximum internal energy during the American ginseng main root collision are positively correlated with the drop angle. After the American ginseng main root rebounds, residual stress and energy remain within it.
3. The bruise volume of the American ginseng main root on different contact materials increased with the drop angle, with the order of bruise volume being: steel > wood > PVC > rubber. At the same drop angle, the bruise resistance index of rubber was higher than that of the other three materials.
4. This study established a predictive model for the bruise resistance index of the American ginseng main root using finite element simulation technology, providing a scientific basis for the design and optimization of harvesting machinery and its key components, with the objective of effectively controlling the American ginseng bruising problem.

## Data Availability

The original contributions presented in the study are included in the article/supplementary material. Further inquiries can be directed to the corresponding authors.
